# MicroRNAs as predictive biomarkers of treatment response to tyrosine kinase inhibitors in hepatocellular carcinoma: how much is missing?

**DOI:** 10.18632/oncoscience.564

**Published:** 2022-09-15

**Authors:** Laura Gramantieri, Francesca Fornari

**Keywords:** HCC, microRNA, biomarkers, TKI

Hepatocellular carcinoma (HCC) is the second leading cause of cancer-associated mortality, with a poor prognosis when diagnosed at advanced stages. Despite the advent of immune-checkpoint inhibitors [1], a paradox is being experienced for systemic treatment of HCC in the personalized medicine era. On one side, randomized clinical trials led to the approval of multiple therapeutic approaches whereas, on the other side, the absence of predictive biomarkers for patient allocation hampers the full potential of personalized medicine. AFP remains the only predictive biomarker of treatment response. Indeed, the phase III clinical trials that investigated ramucirumab as a second line agent after sorafenib progression reported a significant survival benefit only in HCC patients with baseline AFP ≥400 ng/mL [2].

The high stability of microRNAs (miRNAs) in body fluids [3] and the technical easiness of their quantification [4] make them ideal circulating biomarkers. Nevertheless, several methodological issues remain to be addressed before circulating miRNAs can enter the clinical practice. For example, low concentration of certain miRNAs in the bloodstream, contamination from hemolyzed samples, different housekeeping genes and extraction techniques, different platform/assays, heterogeneous control groups (healthy versus cirrhotic patients) and different sample processing (serum versus plasma versus exosomes) limit the comparisons among studies and the identification of informative miRNAs.

We previously demonstrated that serum levels of miR-221 help to discriminate responder and non-responder patients when quantified before sorafenib treatment. In line with miR-221 oncogenic functions, high miRNA serum levels predicted sorafenib resistance in HCC patients. Notably, sorafenib treatment increased circulating miR-221 levels in responder patients over time, suggesting the release of this oncomiRNA from the intra-cellular compartment as an event associated with treatment response. Indeed, miR-221 decrease in HCC cells leads to the re-activation of tumor suppressor genes such as CDKN1B/p27, CDKN1C/p57, PTEN and CASP3 just to name a few [5]. Similarly, we reported higher miR-30e-3p serum levels in patients non-responding to sorafenib with respect to responding ones when monitored two-months after treatment, suggesting its role in predicting early tumor escape [6]. In this regard, Teufel et al. identified nine plasma miRNAs (MIR30A, MIR122, MIR125B, MIR200A, MIR374B, MIR15B, MIR107, MIR320, MIR645) as predictive of improved overall survival (OS) to the second-line agent regorafenib. In particular, increased levels of another member of the miR-30 family, miR-30a, associated with regorafenib benefit when evaluated at the baseline in HCC patients following sorafenib progression [7]. Interestingly, a functional analysis of hypothetic target genes of these miRNAs revealed a possible association between regorafenib response and well-differentiated, hepatocyte-like HCC subtypes (Hoshida S3 subclass).

MiR-181a-5p was identified as a serum biomarker predictive of early sorafenib response in HCC patients and the only independent factor for disease control and OS when analyzed before treatment [8]. A recent study reported the association between high miR-10b-3p serum basal levels and OS, but not progression-free survival (PFS), in sorafenib-treated HCC patients. An increase of miR-10b-3p in the low-level group after four weeks of sorafenib treatment agreed with a better response, paving the way for further investigations in a larger patient cohort [9]. Specifically, miR-10b-3p decreased in both tissues and sera of xenograft animals implanted with Huh7 cells resistant to sorafenib with respect to sensitive ones. Cyclin E1 targeting by miR-10b-3p was demonstrated as a molecular mechanism involved in cell aggressiveness and sorafenib resistance. In this regard miR-518d-5p, a member of the C19MC, showed higher levels in both tissue and serum specimens from HCC patients. In BCLC-C patients, high basal miR-518d-5p correlated with a shorter treatment duration and OS, suggesting its possible employment as a predictive biomarker of sorafenib resistance to be used in the setting of treatment choice. As for other C19MC, miR-518d-5p overexpression in cancer cells blocked mitochondrial-dependent apoptotic cell death through c-jun/PUMA targeting and enhanced sorafenib resistance by metabolic shift and by limiting oxidative stress and ROS production [10]. An interesting retrospective study in HBV-related HCCs that underwent sorafenib treatment documented the predictive potential of the hepato-specific miR-122 whose high basal levels correlated with increased PFS and OS at 12 and 24 weeks after treatment, whilst the low-level group associated with higher AFP and multifocality. The innovative aspect of this study regards the sub-analysis within the high HBV-load group that showed the loss of the relationship between miR-122 levels and OS, suggesting viral load as a confounding factor to the identification of predictive biomarkers [11].

To sum up, circulating miRNAs hold promise as predictive biomarkers to support therapeutic decisions and personalized medicine. Further studies carried out with standardized analytical methodologies in larger patient cohorts with robust subgroup analyses might strengthen present findings (Table 1) and lead to the identification of new candidates.

**Table 1:** List of microRNAs with a predictive role in HCC

MIRNA	Therapeutic treatment	Sample	Baseline/on treatment analysis	MiRNA levels in patients with better response
miR-221	sorafenib	serum	baseline	lower
miR-221	sorafenib	serum	on treatment	higher
miR-30e-3p	sorafenib	serum	on treatment	lower
miR-30a	regorafenib	plasma	baseline	higher
miR-122	regorafenib	plasma	baseline	higher
miR-125b	regorafenib	plasma	baseline	higher
miR-200a	regorafenib	plasma	baseline	higher
miR-374b	regorafenib	plasma	baseline	higher
miR-15b	regorafenib	plasma	baseline	lower
miR-107	regorafenib	plasma	baseline	lower
miR-320	regorafenib	plasma	baseline	lower
miR-645	regorafenib	plasma	baseline	lower
miR-181a-5p	sorafenib	serum	baseline	higher
miR-10b-3p	sorafenib	serum	baseline	higher
miR-10b-3p	sorafenib	serum	on treatment	higher
miR-518d-5p	sorafenib	serum	baseline	lower
miR-122	sorafenib	serum	baseline	higher

The knowledge of molecular drivers of deregulated miRNAs, downstream pathways and feedback loops in which miRNAs are entangled, together with the characterization of cell types acting as producers of extracellular miRNAs and, at the same time, as targets of aberrantly expressed miRNAs would be pivotal to define the settings in which each miRNA might be more informative. Moreover, preclinical studies suggest that instead of a “one fits all” approach, interrogation of miRNAs in defined settings might add a relevant contribution to answer specific questions.
